# How is perceived community cohesion and membership in community groups associated with children’s dietary adequacy in disadvantaged communities? A case of the Indian Sundarbans

**DOI:** 10.1186/s12913-016-1862-z

**Published:** 2016-11-15

**Authors:** Debjani Barman, Lalitha Vadrevu

**Affiliations:** IIHMR University, Jaipur, India

## Abstract

**Background:**

Membership in community groups and a sense of community cohesion may facilitate collective action in mobilizing resources towards better health outcomes. This paper explores the relationship of these factors, along with individual level socio-economic variables, to dietary adequacy among children below 6 years of age, a proximate determinant of child malnutrition.

**Methods:**

We conducted a cross-sectional survey in Patharpratima block of the Sundarbans in West Bengal, India, using a two-stage, 30 cluster random sampling design. In 1200 sampled households, we used a structured questionnaire to interview mothers of children below 6 years of age on their child’s nutritional intake. We also interviewed household heads to assess perceived community cohesion using a nine item scale, membership in any community self-help organization, and other socio-economic determinants. We used a logistic regression model to assess their association with a minimum acceptable diet among children between 6 months to 6 years.

**Results:**

Only 9.33 % children between 6 and 71 months of age received a minimum acceptable diet. With each increase in the perceived community cohesion score (scale 0-9), a child is 1.31 times more likely to have minimum acceptable diet (95 % CI 1.14, 1.50). The odds of minimum acceptable diet were also higher among children whose mothers had primary education (2.09, 95 % CI 1.03, 2.94) as compared to illiterate mothers and in households with surplus food resources (2.72, 95 % CI 1.32, 5.58) as compared to those without surplus or deficit. In contrast, registering at an Anganwadi (government early child development) centre (odds ratio 1.34 95 % CI 0.69, 2.60) and community membership (odds ratio 0.93, 95 % CI 0.59, 1.46) were not associated with minimum acceptable diet.

**Conclusion:**

The results are consistent with what is known about the importance of maternal education and access to food resources in ensuring that children have a minimum acceptable diet. Perceived community cohesion seems to play a positive role in children’s diets. Further research needs to clarify which community characteristics and services are the most relevant, how they can better support children’s diets, and how interventions can strengthen these community characteristics and services.

## Background

Malnutrition is an issue of tremendous concern especially in countries such as India where almost 40 % of the children are underweight [1, 2]. One of the proximate and critical determinants of malnutrition is dietary adequacy [3]. Children need adequate nutrition of acceptable quality and quantity to prevent malnutrition. So far programs on malnutrition in India have extensively focused on improving feeding practices and access to supplementary nutrition of mothers and children below 6 years of age. But adequacy of nutritional intake still remains a concern.

According to CARE’s Infant and Young Child Feeding Practices (IYCF), minimum acceptable diet is a composite indicator that includes both minimum dietary diversity and minimum meal frequency for 6–23 months children after factoring in child breast feeding status [4]. An analysis of India’s National Family Health Survey data revealed that dietary diversity was one of the five most important predictors of childhood stunting/underweight in India [5]. A similar association was found by Fenske et al. where food diversity and meal frequency in a household had a linear positive association by age on stunting in children below 5 years of age in India [6]. Social and economic factors at the individual, household and community level [7, 8], including household food security [2, 9], income [10, 11], occupation (e.g. agricultural dependence [7]), and educational status of the caregivers [12], especially of mothers, have an immediate bearing on the child’s nutritional intake. In India, an analysis of country level data found that nutritional adequacy measured through minimum acceptable diet, was determined by mothers’ education, utilization of maternal health services and household income [13].

It is widely acknowledged that along with demographic and economic factors, people and communities play a crucial role in safe guarding health [14, 15]. The Alma Ata declaration in 1978 enshrined this role, calling for “maximum community and individual self-reliance and participation in the planning, organization, operation and control of primary health care, making fullest use of existing resources” [15]. In the field of child nutrition, there has been a shift from centrally planned programs to community-based approaches, recognizing that community engagement is vital for the design, uptake, scalability and sustainability of health interventions [16–18]. Ramalingaswamy et al. in their commentary exploring the roots of malnutrition argue that the problem of malnutrition is rooted in social inequities where communities not only need access to basic amenities but also the information, confidence and support to translate access to services into a reduced burden of disease [19]. Inspite of the importance of community participation for health, there is a lack of understanding of the attributes and capacities of a community that can facilitate health action [20].

Goodman et al. define “community capability” has a composite of community leadership, skills, resources, organizational networks, sense of community, understanding of community history, power and values among other aspects [21]. The term “community capability” encompasses key concepts of empowerment, mobilisation, social capital and capacity building’ [22]. Instruments to quantitatively measure community capabilities have focused on a range of dimensions, including: groups and networks; trust and solidarity; collective action, cooperation and participation; information and communication; social cohesion and inclusion; empowerment and political action; and community autonomy [23–25].

This study is part of the Future Health Systems Research Programme Consortium, which defined community capability as the combined influence of a community’s social systems and collective resources that can be applied to address community problems and broaden community opportunities [26]. In the present, article we focus on two specific dimensions of community capacity: household head’s membership in community organizations and perceived community cohesion and explore its association with children’s nutritional adequacy.

The importance of membership in groups and perceived community cohesion is widely explored with respect to child health outcomes like malnutrition. Evidence suggests that individuals by virtue of their membership in various social groups like self-help groups, occupational or religious groups and other social organizations, have the capacity to command scarce resources for improving health outcomes like nutrition. Community organizations facilitate information sharing and collective decision making and reinforces positive behaviours and practices [27]. Specifically, the mother’s membership in community organizations is found to be an effective instrument against undernourishment for those children born to socio-economically disadvantaged mothers, as they can better access community and child health related information and resources [28]. De Silva et al. in an analysis of cross-sectional data from Peru, Ethiopia, Vietnam and Andhra Pradesh in India, found that community membership in more than one organization was negatively associated child malnutrition [29]. Tripathy et al report that memberships in self-help groups show a positive effect on neonatal mortality rate by mobilizing communities towards collective action [30].

In terms of mechanisms that explain the link between community cohesion and nutrition, research has shown that membership in community organizations and community cohesion affect child malnutrition by either improving mother’s social interaction, thus improving information sharing and knowledge; reinforcing positive social behaviours that increases cohesiveness, or by explicit coordination where mothers provide support to one-another in child care practices [31]. Havemann in a case study on the effect of community based social program intervention on malnutrition found that more cohesive communities were more able to develop and implement their plans [32].

Although some research points to an association between membership in community groups, social cohesion and better nutrition; there is a gap in literature on the association of these attributes and capacities on nutrition related practices like feeding and diet for children [28, 29, 33]. In the present article we hypothesize that membership in community organizations and perceived community cohesion are associated with child’s dietary adequacy in disadvantaged communities like the Indian Sundarbans, a predominantly rural, poor and agrarian region in the state of West Bengal, India.

## Methods

### Context – Sundarbans of West Bengal

The Indian Sundarbans is a mangrove delta located on the Bay of Bengal in the state of West Bengal in the north-eastern part of India. The Sundarbans is intersected by tidal creeks and rivers, making it highly inaccessible and susceptible to climatic shocks and recurrent floods. In the study area as many as 70 % of households had faced at least one natural calamity in the last five years preceding the survey. These climatic events have also severely damaged community infrastructure such as roads and health facilities.

The Sundarbans population is diverse, with people from many ethnic, religious and occupational groups co-existing together. Historically, the region was occupied by migrants and refugees from India and Bangladesh who arrived during India’s independence and during the India-Pakistan war of 1972. In terms of child health, the burden of malnutrition and child morbidity in the Sundarbans is higher than the West Bengal average [34].

There are several types of community organizations in the Sundarbans, including farmers groups, youth clubs, self-help groups and fishermen clubs. Self-help groups form one of the largest types of social groups in states like West Bengal; as of 2009 there were over 7000 in the Sundarbans [35]. Self-help groups arose from a social movement for economically empowering women and men by building their capacity, providing infrastructural support for income generation activities, extending micro-credit support [36].

**Fig. 1:**
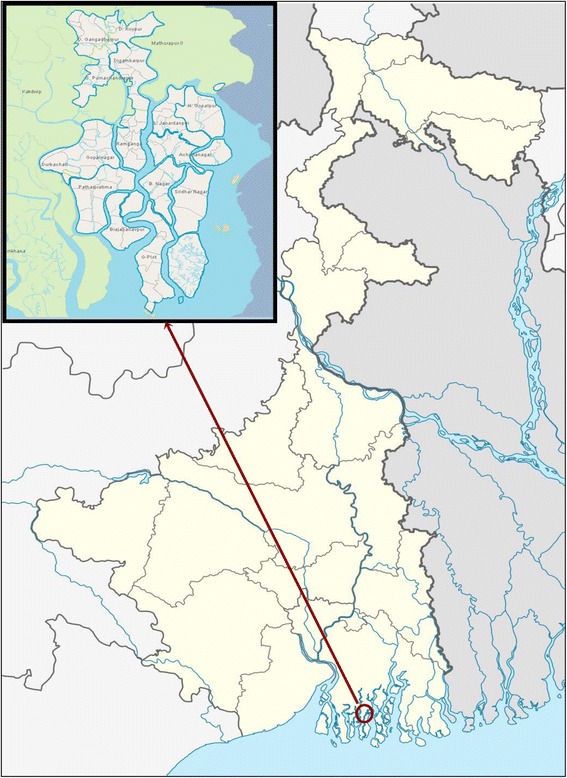
Study area in the Indian Sundarbans

The present study was conducted in the Patharpratima block of the Sundarbans (see Fig. 1), which has a population of approximately 331,000 people spread over 15 g Panchayats (sub-block level administrative units) and 87 villages. According to census 2011, the block records a literacy rate of 72 % [37]. People living in the block are generally of poor economic status. Marginalized community groups (scheduled castes, scheduled tribes and religious minorities) comprise almost 40 % of the population. According to a rural household survey conducted in 2005, a little over half of the households in the block are from Below Poverty Line (BPL) category [38]. There are hardly any employment opportunities outside the primary sector (agriculture, forestry, fishing and mining) and two-thirds of the working population is associated with agriculture.

### Study design

We base the present article on primary data collected from Patharpratima block in the South 24 Paraganas district of the Indian Sundarbans during April and May 2012. We used a two-stage stratified sampling technique for selecting the respondents. An earlier Future Health Systems study divided the region into poor, moderate and good blocks based on physical accessibility and service delivery [34]. Out of the six poor blocks, Patharpratima was selected for this present study given its mixed geographical characteristics in the form of deltaic (river locked) and non-deltaic (land locked region) and demographic composition. The complex terrain and proximity to the ocean makes populations in this region especially vulnerable to floods. In the first stage, we classified the block’s 15 g Panchayats in to two strata, deltaic and non-deltaic, to ensure representation from both geographically accessible and inaccessible regions. In the second stage, we selected 30 villages using probability proportional to size. We then sampled 40 households with at least one child aged 0–6 years from each village, for a total of 1200 households. We calculated the sample by considering the prevalence of malnutrition in the region.

### Dependent variable

To assess child diet, we interviewed the mother or caregiver of the youngest child (0–6 years) in the sampled household using a structured interview schedule. We used the minimum acceptable diet as an indicator of adequate nutritional intake, following the Infant and Young Child Feeding (IYCF) Practices guideline by CARE 2010 [4]. We followed the guidelines specified by IYCF for 6–23 months age group. For 24–71 months children, we first calculated the minimum dietary diversity following the Food and Agriculture Organization (FAO) 2013 guideline and minimum meal frequency by extending the criteria under IYCF to children above 23 months of age [39]. We assumed that an average child above 23 months of age, will need at least four meals a day irrespective of breastfeeding status.

We asked mothers or caregivers if the youngest child consumed any of the following food items: grains, white tubers and roots, vegetables, fruits, meat, eggs, fish and other sea food, legumes, nuts and seeds, milk and milk products, oils and fats in the last 24 h (Table 1). The responses were segregated in to eight food groups according to IYCF and FAO. We measured minimum dietary diversity as the total count of different food groups consumed by the child as mentioned in details in Table 1. We coded a child as having a value for minimum acceptable diet as ‘1’, if they received minimum acceptable diet appropriate for their age in the last 24 h and rest of the cases were coded as ‘0’.

**Table 1:** Construction of the outcome variable: Minimum Acceptable Diet

Age of the Child in months	Minimum dietary diversity as per IYCF or FAO	Minimum Meal frequency as per IYCF	Minimum acceptable diet as per IYCF	Minimal acceptable diet used in the paper
6–8 month breastfeeding	Minimum of 4 or more food groups out of seven food groups like-Grains, roots and tubers, Legumes and nuts, Dairy products (milk, yogurt and cheese), Flesh foods (meat, fish, poultry and liver/organ meats), Eggs, Vitamin-A rich fruits and vegetables, Other fruits and vegetables	2 times	*Breastfed* children 6–23 months of age who had the minimum dietary diversity and the minimum meal frequency during the previous day	Children 6–71 months of age who had at least the minimum dietary diversity (minimum four or more food groups following different food groupings based on their age) and the minimum meal frequency (different frequency as per age) during the previous day
9–23 months breastfeeding		3 times		
6–23 month non breast feeding		4 times	*Non-breastfed* children 6–23 months of age who had at least the 2 milk feedings and had at least the minimum dietary diversity and the minimum meal frequency during the previous day	
24–71 months	Minimum of four or more food groups like Cereals, roots and tubers, vitamin A rich fruits and vegetables, Other fruits, Other vegetables, Legumes, pulses and nuts, Oils and Fats, Meat, poultry, fish, Dairy, Eggs and Other (sweets, chips, soda, condiments) etc	4 times (no guideline available)	No guideline available	

### Independent variables

We interviewed the head of the household to assess the independent variables: membership in community groups, perceived community cohesion and household characteristics. For measuring community membership we asked the head of the household if he/she is a member of any community groups.

We measured perceived community cohesion using the community cohesion questionnaire of the Unlocking Community Capabilities Instrument. Respondents were read nine statements and asked to reply on a four point likert scale from strongly disagree to strongly agree (Table 2).

**Table 2:** Nine-item questionnaire to assess perceived community cohesion

Number	Statement to assess perceived community cohesion
1	As members of this community we are all committed to the same collective goals
2	whenever our community undertakes an objective, all work hard until it is accomplished
3	I am confident that we as community members can develop and carry out solutions to problems as they arise
4	People with differing views are able to equally contribute their views on community plans and activities
5	People from different economic status in this community are able to equally contribute their views on community plans and activities
6	Women in this community are able to equally contribute their views on community plans and activities
7	When conflicts or disagreements arise between community members, other community members get involved for resolving the issue
8	Community leaders listen to input from everyone within the community when making a decision
9	Community leaders represent the interests of weaker people and women in this community

The non-standardized variables were used and reduced using principal component analysis to explore the underlying constructs for these components of community capability. All the variables clustered in to logically coherent latent constructs that could potentially represent aspects of equality, unity and participation. All constructs were perceived to be important elements of community capability and all nine questions warranted inclusion in the analysis. The responses were clustered on the centre on the likert scale so each response was recoded to a dichotomous variable for simplicity. Thus, the responses ‘agree’ and ‘strongly agree’ were coded to ‘0’ and ‘disagree’ and ‘strongly disagree’ to ‘1’. The household perceived community cohesion score was calculated by summing up the study variables, where the possible score could range from 0 to 9.

Heads of households were asked a standard set of questions to assess the following household characteristics: location (delta/non-delta), religion, caste, access to drinking water, access to toilet, perceived food security, mother’s education, mother’s age, child’s age, child’s sex. Mother or care giver of the child were asked questions on child’s place of birth (institutional or home) and child’s registration at Anganwadi Centers (government child development centers).

We categorized household’s perceived food security status into three categories: (1) Not adequate food throughout the year/few months of the year; (2) neither deficit nor surplus; and (3) surplus food throughout the year. We categorized mother’s age into three categories: 16–25 years, 26–29 years and 30 years and above. Mother’s education was categorized into: (1) illiterate, (2) upto primary and (3) upto secondary or higher. We categorized caste into two categories: (1) general caste and (2) scheduled caste (SC) or scheduled tribe (ST) or other backward class (OBC)).

The study was approved by the Institute’s ethical review board in 2012. All respondents were interviewed only upon obtaining written informed consent.

### Statistical analysis

Since complementary feeding other than breast milk should be introduced at six months of a child, the analysis was carried out with children aged six months and above. Therefore out of total sampled children, the analysis has been carried out on 922 children only. To explore the association of minimum acceptable diet with its determinants, we used logistic regression. Sample error was calculated by adjusting for clustering at the village level. We carried out the data analysis in STATA 11 [40].

## Results

### Sample characteristics

Only 9.33 % of the children between 6 and 71 months of age received a minimum acceptable diet (Table 3). Almost all children (97.18 %) consumed grains in the 24 h recall period, but only 42.08 % of children consumed the next most popular food group, vitamin A rich fruits and vegetables. The mean meal frequency was 3.50 meals per day and showed an increase with age (Table 3). Despite the low levels of minimum acceptable diet, the majority of households (71.26 %) reported neither surplus nor deficit of food in terms of household's ability to meet the annual food requirement (Table 4).

**Table 3:** Children’s dietary diversity and meal frequency

Dietary diversity food groups	Percentage of children having food from the group (%)
Grain, roots, tubers	97.18
Vitamin A rich fruit and vegetable	42.08
Flesh food	38.50
Dairy products	19.96
Other fruit and vegetable	19.09
Legumes & nuts	11.93
Egg	4.34
Oil and fats	0.43
Meal frequency	Mean number of meals per day (standard deviation)
Children 6–8 months breastfeeding	3.41 (1.18)
Children 9–23 months breastfeeding	3.41 (1.18)
Children 6–23 months non breastfeeding	3.71 (1.08)
Children more than 23 months	3.71 (1.01)
Children 6–71 months	3.50 (1.15)
Children 6–71 months with minimum acceptable diet (%)	9.33

**Fig. 2:**
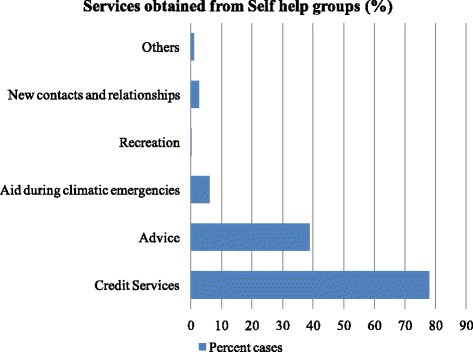
Percentage distribution of services received from self help groups

With regards to individual characteristics, the majority of the mothers (66.27 %) were educated up to primary level, while 18.55 % of mothers were illiterate. Fifty- nine percent of the mothers belonged to the first age group 16–24 years. 72.56 % of children belong to general caste. Half of the sampled children (51.95 %) were delivered at home and 84.60 % children were registered at Anganwadi centres. The mean age of the sampled children was 32.33 months. With regards to community characteristics, fewer than half (44.79 %) of the heads households reported being members of at least one community group. Among those who were members of a community group, 85 % are members of a self-help group. Households primarily relied on self-help groups for financial credit (Fig. 2).

Table 4 represents the distribution of the background characteristics by the outcome variable, minimum acceptable diet. The mean perceived community cohesion score, out of a possible maximum score of nine, was 5.41 for children who received minimum acceptable diet and 4.57 for children who did not receive minimum acceptable diet and the difference between these two groups was statistically significant (*p* = 0.000). The percentage of the heads households reporting being members of at least one community group did not differ significantly (*p* = 0.90) between household where the children received and did not receive minimum acceptable diet. The percentage of children with minimum acceptable diet was higher in households with surplus food resources (*p* = 0.54), among children born to more educated mothers (*p* = 0.50), female children (*p* = 0.31), children born in an institution (*p* = 0.54), and children registered at an Anganwadi center (*p* = 0.31), although none of these differences were significant (Table 4).

**Table 4:** Background Characteristics of children by status of Minimum Acceptable Diet

	Minimum acceptable diet		Chi 2 Value (*p*-value)	*T* test value (*p*-value)
	Yes	No		
*N* (%)	86 (9.33)	836 (90.67)		
Community cohesion *N* (Mean, standard deviation)	86 (5.41, 1.78)	836 (4.57,1.71)	-	−4.29 (0.000)
Membership in Community Groups *N* (%)				
Yes	38 (9.20)	375 (90.80)	0.01 (0.90)	-
No	48 (9.43)	461 (90.57)		
Perceived food security status *N* (%)				
Not adequate food throughout the year/some months of year	20 (9.17)	198 (90.83)	0.37 (0.54)	-
Neither deficit nor surplus	56 (8.52)	601 (91.48)		
Surplus food throughout the year	10 (21.28)	37 (78.72)		
Mother’s Education *N* (%)				
Illiterate	8 (4.68)	163 (95.32)	5.99 (0.50)	-
Up to Primary	61 (9.98)	550 (90.02)		
Secondary & above	17 (12.14)	123 (87.86)		
Mother’s age in years *N* (%)				
16–25	51 (9.34)	495 (90.66)	0.51 (0.78)	-
26–29	22 (10.23)	193 (89.77)		
30-Max	13 (8.07)	148 (91.93)		
Child’s gender *N* (%)				
Male	42 (8.43)	456 (91.57)	1.02 (0.31)	-
Female	44 (10.38)	380 (89.62)		
Age in completed months *N* (mean, standard deviation)	86 (32.19,13.13)	836 (32.34, 17.40)	-	0.08 (0.94)
Caste *N* (%)				
General	67 (10.01)	602 (89.99)	1.36 (0.24)	-
SC/ST/OBC	19 (7.51)	234 (92.49)		
Place of delivery *N* (%)				
Home	42 (8.77)	437 (91.23)	0.37 (0.54)	
Institution	44 (9.93)	399 (90.07)		-
Child registered at Anganwadi center *N* (%)				
Yes	76 (9.74)	704 (90.26)	1.04 (0.31)	
No	10 (7.04)	132 (92.96)		

### Regression result

Table 5 depicts the result of logistic regression. With each one point increase in perceived community cohesion score, a child was 1.31 times more likely to have minimum acceptable diet (OR: 1.31, 95 % CI 1.14–1.50). Children from household with memberships in at least one organization were more likely to have minimum acceptable diet but the result was not statistically significant. With the increase in perceived household food security child was 2.72 times more likely to have minimum acceptable diet and the result was statistically significant (95 % CI 1.32–5.58). Compared to children of illiterate mother, children of mother with primary (OR: 2.09, 95 % CI 1.03–4.24) and secondary and more education (OR: 2.27, 95 % CI 0.92–5.60) were more likely to have minimum acceptable diet diversity but the result was statistically significant only in the first case when mother was educated up to primary level. Mother’s age, place of delivery, child’s sex and Anganwadi center registration had no statistically significant association with minimum acceptable diet. To test multicollinearity variance inflation factor was calculated for all the predictors of regression model and the analysis negates the possibility of multicollinearity.

**Table 5:** Odds ratio of predictor variables of Logistic Regression on Minimum Acceptable Diet

	Final model		
	Odds ratio	CI (95 %)	
Community cohesion	1.31	1.14	1.50
Membership in community groups: No membership (Ref)	0.93	0.59	1.46
Perceived household food security Status: Neither deficit nor surplus (Ref)			
Not adequate food throughout the year	1.27	0.81	2.02
Surplus food throughout the year	2.72	1.32	5.58
Mother’s Education: Illiterate (ref.)			
Up to Primary	2.09	1.03	4.24
Secondary & above	2.27	0.92	5.60
Mother’s Age: 16–25 years (ref.)			
26–29	1.09	0.60	1.98
30-Max	0.90	0.45	1.80
Gender: Female (ref.)			
Male	0.81	0.52	1.26
Age (mean) in completed months	1.00	0.99	1.01
Caste General (ref.)			
SC/ST/OBC	0.80	0.40	1.59
Place of Delivery: Home delivery (ref.)			
Institution	0.91	0.61	1.37
Registration with Anganwadi centre Children not registered (ref.)			
Anganwadi centre	1.34	0.69	2.60
Prob > chi2			0.0001
Pseudo R2			0.0559
Log likelihood			−269.88

## Discussion

Our study finds that a child’s nutritional adequacy measured as minimum acceptable diet is associated with the household’s perceived community cohesion score, mother’s education status and household’s perceived food security. We find no statistically significant association between minimum acceptable diet and membership in community groups, sex of the child and utilization of Anganwadi Centre services.

One probable reason why membership in community groups does not show any association with the minimum acceptable diet in our study is that majority of the households are mainly members of self-help groups, which are primarily used for credit services during emergencies and not for any explicit health related activities. Other studies have found that membership has a protective effect against child malnutrition [29]. Memberships in community groups can facilitate peer support in which individuals gain access to information, emotional support and gain competencies [41]; perhaps self-help groups in the region we studied are not providing this degree or type of social support.

Community cohesion is reflected in the degree of trust and reciprocity among community members that can facilitate support and collective action. It is also a critical determinant of resilience in communities [42] and is a strong predictor of positive social and health behaviours [43]. The positive association of perceived community cohesion with child nutritional intake suggests that living in a community where members participate and engage in various social activities and collective decision making can have a positive effect on child feeding. This finding underlines the need for policymakers and program managers to consider community context when designing interventions for improving child nutritional intake.

We found no statistically significant difference between minimal acceptable diet by child gender, which echoes the findings of other studies on child feeding in South Asia [13, 44, 45]. However, given high levels of male child preference in the region, we expected boys to be significantly more likely to receive minimum acceptable diet than girls, when in fact our (non-significant) findings show that girls were more likely to receive minimum acceptable diet than boys. A possible reason behind this could be the extended periods of breastfeeding among male children compared to female children. The result from a scoping study on maternal and child health in the block by Kanjilal et al. find that ‘About two-thirds of male children and 77 % of females received complementary food on time. A male child, if exclusively breastfed for 5 months, is more likely to continue receiving exclusive breastfeed even beyond the period than a female child is even though he needs complimentary foods [46].

In our study more educated mothers were more likely to feed their children with minimum acceptable diet. This is in line with previous studies from south east Asian contexts that show a positive association of a child’s nutritional intake with mother’s educational status [13, 44, 45, 47, 48]. Analysis of the Indian National Family Health Survey has similarly found mothers education (and household income) to be important predictors of child feeding practices [48]. The positive association between perceived household food security and child’s nutritional intake is also expected, and aligns with other research linking poverty to poor dietary diversity in low and middle income countries [49]. Poor households have been found to depend largely on starchy staples for their diet, with hardly any animal products, fruits or vegetables [49].

Although our study tries to explore the association between nutritional adequacy and community’s capability, the results need to be interpreted keeping in view some study limitations. We have not used some important variables including: direct measures of economic status (income or wealth index), child malaria, tuberculosis, bottle feeding during the day and information on mothers’ membership in community groups, due to lack of data. For example, membership of mothers in community groups that work in specific health related activities might have positive impact on child feeding, as compared to membership of the head of the household in groups that provide credit services. It is advisable that for understanding the associations and linkages between community capabilities and child health, mothers’ access to community groups and resources should be included in the measurement of households’ community capabilities. Also, further research should focus on a deeper exploration of communities’ capability in a given context and its linkages with child health outcomes.

Our study tries to measure community cohesion as perceived by heads of households along with other socio-economic factors. Further research in the area could adopt both quantitative and qualitative measures to comprehensively explore and understand community capability to collectively address health and child nutrition issues. Extensive literature exists on the determinants of child feeding practices and child nutritional outcomes (stunting, underweight and wasting), yet, there is a gap in evidence exploring the association between community level attributes like community cohesion, and memberships on these outcomes. Our results serve as a catalyst to further research in this area.

## Conclusions

Although there is a wide network of government nutrition programs in the Sundarbans, malnutrition remains a persistent issue. Prior research suggests that many programs that failed to demonstrate impact faced challenges in terms of community participation, involvement and interaction. It is vital that programs seeking to counter malnutrition engage with communities’ capabilities. Community cohesion and membership in community groups are an important aspect of communities’ capabilities. This study underlines the importance of these community level factors as potentially playing a role in children’s diets. Programs that include community participation and empowerment can explore the usage of quantitative measures, like those used in this article, to gauge the extent to which community capability as an intermediary outcome of community participation and empowerment impacts child health outcomes. Our evidence serves as a primer for future research exploring the concept of community cohesion in the area of child health.

## Abbreviations

CI: Confidence interval
FAO: Food and Agriculture Organization
IYCF: Infant and young child feeding practices
OBC: Other backward class
OR: Odds ratio
SC: Schedule caste
ST: Schedule tribe
